# Fecal Carriage and Risk Factors Associated with Extended-Spectrum β-Lactamase-/AmpC-/Carbapenemase-Producing *Escherichia coli* in Dogs from Italy

**DOI:** 10.3390/ani14233359

**Published:** 2024-11-21

**Authors:** Alessia Facchin, Gabriele Ratti, Joel Filipe, Martina Penati, Alessia L. Gazzonis, Greta Masiero, Paola Dall’Ara, Giovanni L. Alborali, Stefania Lauzi

**Affiliations:** 1 Department of Veterinary Medicine and Animal Sciences, University of Milan, Via dell’Università 6, 26900 Lodi, Italy; 2 Istituto Zooprofilattico Sperimentale della Lombardia e dell’Emilia Romagna “Bruno Ubertini”, Via Bianchi 7/9, 25124 Brescia, Italy

**Keywords:** canine, antimicrobial resistance, ESBL, AmpC, carbapenemase

## Abstract

MDR bacteria are an emerging global threat to public health, and the role of dogs in the rise of antimicrobial resistance is under investigation. This study investigated the fecal shedding of extended-spectrum β-lactamase (ESBL)-, AmpC- and carbapenemase (CP)-producing *Escherichia coli* and associated risk factors in dogs admitted to the Veterinary Teaching Hospital of Lodi, University of Milan, or other veterinary clinics and kennels in Northen Italy. Feces collected in 2020–2022 were microbiologically and molecularly analyzed. ESBL-/AmpC-/CP-producing *E. coli* was detected in 14/100 (14%) dogs. Eleven (11%), five (5%) and one (1%) dogs carried ESBL-, AmpC- and CP-producing *E. coli* phenotypes, respectively, supported by the PCR detection of *bla*_CTX-M_ and/or *bla*_TEM_ in ESBL-producing *E. coli*; *bla*_CMY-2_ and the presence of putative low-level AmpC production in AmpC-producing *E. coli*; and *bla*_OXA-48_ in CP-producing *E. coli*. Different combinations of resistance genes and genetic features were observed. Multidrug resistance was observed in 13/14 (92.9%) *E. coli* isolates. Binary logistic regression analysis showed that ESBL-/AmpC-/CP-producing *E. coli* fecal shedding tended to be associated with antibiotic treatment *(p* = 0.058; OR = 3.87). The detection of ESBL-/AmpC-producing *E. coli*, along with the presence of a carbapenemase-resistant *E. coli* isolate from domestic dogs, although still limited, emphasizes the need for antimicrobial stewardship and specific surveillance programs, particularly for CP-producing bacteria in companion animals.

## Simple Summary

Antimicrobial resistance is a global health threat because microorganisms causing infections no longer respond to antimicrobial medicines, and this is particularly important in the case of resistance to last-resort antibiotics such as carbapenems. The role of dogs in the rise of antimicrobial resistance is under investigation. This study investigated the presence of *Escherichia coli* (*E. coli*) showing specific antimicrobial resistance patterns—defined as extended-spectrum β-lactamase (ESBL), AmpC and carbapenemase (CP) production—in dogs’ feces from Northern Italy. Out of 100 samples, 14 (14%) dogs showed the presence of these resistant *E. coli*, with one (1%) bacterium showing resistance to carbapenems. Different combinations of resistance patterns were observed in these 14 *E. coli*. The majority (13/14, 92.9%) of the *E. coli* were also resistant to three or more different classes of antibiotics and were classified as multidrug-resistant. The presence of the resistant bacteria in feces tended to be associated with antibiotic treatment. The identification of bacteria known as ESBL-/AmpC-producing *E. coli* in domestic dogs, along with the presence of a bacterium resistant to carbapenems, although still limited, emphasizes the need for the adequate control of antimicrobial therapy and administration of antibiotics and surveillance programs for carbapenem-resistant bacteria in companion animals.

## 1. Introduction

Antimicrobial resistance (AMR) is one of the world’s most complex challenges. The World Health Organization (WHO) has reported that the emergence and rapid spread of AMR is expected to cause 10 million deaths per year by 2050, surpassing diabetes, heart disease, and cancer as the leading cause of death in humans [1,2,3,4].

Extended-spectrum cephalosporin-resistant *Enterobacteriaceae* (ESCRE) are considered one of the most important groups of bacteria contributing to the complexity of AMR [5]. Among ESCRE, multidrug-resistant (MDR) *Escherichia coli* has been isolated from humans, animals and the environment worldwide [6]. Resistance patterns of major importance in MDR *E. coli* are extended-spectrum β-lactamase (ESBL) and AmpC beta-lactamase (AmpC) [5,6]. ESBL-/AmpC-producing *E. coli* have been reported as significant threats in both human and veterinary medicine [3,5,7]. The role of food-producing animals [8] as well as companion animals [3,7,9,10] as carriers of ESBL-/AmpC-producing *E. coli* has been reported.

More recently, the emergence of carbapenemase (CP)-producing bacteria has been reported as a serious public health problem. CP resistance is increasing, despite this type of resistance in *E. coli* being less frequently reported compared to ESBL/AmpC resistance patterns [9,11]. The presence of CP-producing *E. coli* has been reported not only in food-producing animals but also in companion animals. Hospitalization and antimicrobial treatment have been reported as risk factors associated with the presence of ESBL-/AmpC-/CP-producing *E. coli* in pets [7,9,10]. Dogs may act as reservoirs of resistance genes, contributing to their distribution and even transmission between humans and animals [9,12]. Consequently, there is the need to intensify surveillance programs for the presence of CP-producing bacteria in pets, in order to formulate measures to prevent their potential spread [11,13].

Considering the importance of AMR monitoring and the high number of pet dogs in Italy, the aim of this study was to evaluate the presence of fecal carriage, the antimicrobial resistance phenotypes and genotypes, and the risk factors associated with ESBL-/AmpC-/CP-producing *E. coli* in dogs.

## 2. Materials and Methods

### 2.1. Animals and Samples Collection

Fecal samples were collected from dogs admitted to the Veterinary Teaching Hospital (VTH) of Lodi or to other veterinary clinics or kennels from the provinces of Pavia and Monza Brianza, Northern Italy, between May 2020 and March 2022.

The Institutional Animal Care and Use Committee and the Institutional Ethical Committee of University of Milan approved the study protocol (OPBA 40/2020).

Information regarding sex, age, season of sampling, type of ownership, health status, hospitalization, collection of samples on admission or during hospitalization, administration of antimicrobial therapy within three months from sampling collection and the administered antimicrobial classes were collected for each animal. Dogs were categorized into two age groups: ≤2 years old and >2 years old, according to previous reports [14]. The clinical status was categorized in the groups of healthy or unhealthy dogs, according to the presence/absence of a clinical manifestation and the results of routine laboratory analyses, if available. Unhealthy dogs showed gastrointestinal, respiratory, dermatological, neurologic, traumatic or other clinical presentations on admission, whereas healthy dogs were apparently healthy.

Fecal samples were stored for a maximum of 4 days at 4 °C or for 1 month at −20 °C and then analyzed.

### 2.2. Isolation and Identification of Escherichia coli

One gram of each fecal sample was diluted in 9 mL of buffered peptone water (BPW) and incubated overnight at 37 °C. A loopful of the culture was then streaked on Ma–Conkey agar supplemented with 1 mg/L cefotaxime [3] and incubated at 37 °C for 24 h. One lactose-fermenting isolate suggestive of *E. coli* (or one lactose-non-fermenting isolate in case of the absence of lactose-fermenting colonies) was selected from each sample and was identified to the species level employing MBT Microflex LT/SH matrix-assisted laser desorption/ionization time-of-flight mass spectrometry (MALDI-TOF MS) (Bruker Daltonik GmbH, Bremen, Germany) and the direct transfer method [15]. *E. coli* isolates identified at the species level were stored at −80 °C in brain heart infusion (BHI) supplemented with 15% glycerol for further phenotypic and genetic characterization, as well as the identification of antimicrobial minimum inhibitory concentration (MIC).

### 2.3. Phenotypical Characterization of ESBL-/AmpC-/CP-Producing E. coli

The *E. coli* isolates were thawed and phenotypically classified according to the guidelines published by the European Committee on Antimicrobial Susceptibility Testing (EUCAST) [16]. Mueller–Hinton agar (MH) plates were inoculated with samples of each isolate adjusted to a turbidity of 0.5 McFarland. The *E. coli* isolates were tested for the confirmation of ESBL production by the combination disk test (CDT). For the detection of AmpC and carbapenemase phenotypes, AmpC detection set D69C and Carbaplus D73C (MAST group Ltd., Bootle, UK) were used, respectively. The Carbaplus D73C (MAST group Ltd., Bootle, UK) set was used to identify KPC, Metallo-β,-lactamase (MBL) or OXA-48 types. Briefly, plates were incubated at 37 °C for 24 h, and the inhibition zone around each antimicrobial disc was interpreted as reported by EUCAST [16] and according to manufacturer’s instructions.

### 2.4. ESBL-/AmpC-/CP-Encoding Genes

*E. coli* colonies with ESBL-, AmpC- and/or CP-positive phenotypic results were resuspended in 50 μL of DNase–RNase-free water, and DNA was extracted by lysing–boiling (98 °C for 10 min) for further molecular characterization. A total of 5 μL of DNA from each isolate was used as a template for the detection of ESBL-, AmpC- and CP-encoding genes according to previously published PCR protocols [17,18,19,20,21,22,23] with minor modifications. For each PCR reaction, we used a 25 μL total reaction volume that included 1*x* Phire Green Hot Start II PCR (ThermoFisher Scientific, Segrate, Milan, Italy) and 30 pm of each primer. The primers used in this study and amplicon lengths are reported in Table S1. More precisely, the ESBL-encoding genes *bla*_CTX-M_, *bla*_TEM_ and *bla*_SHV_ were investigated in *E. coli* isolates, with thermal conditions consisting of initial denaturation at 98 °C for 30 s followed by 35 cycles of 98 °C for 5 s, 60 °C for 5 s and 72 °C for 8 s, and there was a final elongation step at 72 °C for 1 min [17]. For *bla*_CTX-M_-positive samples, the presence of *bla*_CTX-M_ groups 1 or 9, which have been more frequently detected in ESBL-producing *E. coli* in companion animals, was investigated by PCR [18,19]. For *bla*_CTX-M_ group 1, the thermal conditions consisted of initial denaturation at 98 °C for 30 s followed by 35 cycles of 98 °C for 5 s, 50 °C for 5 s and 72 °C for 15 s, and there was a final elongation step at 72 °C for 1 min [18]. For *bla*_CTX-M_ group 9, the thermal conditions consisted of initial denaturation at 98 °C for 30 s followed by 35 cycles of 98 °C for 5 s, 56 °C for 5 s and 72 °C for 15 s, and there was a final elongation step at 72 °C for 1 min [19]. Plasmid-mediated AmpC (pAmpC)-encoding genes investigated for the AmpC phenotype were *bla*_CMY-2_, *bla*_CIT_, *bla*_ACC_, *bla*_FOX_, *bla*_MOX_, *bla*_DHA_ and *bla*_EBC_ [20,21]. The PCR protocol for *bla*_CMY-2_ gene detection consisted of initial denaturation at 98 °C for 30 s followed by 35 cycles of 98 °C for 5 s, 57 °C for 5 s and 72 °C for 13 s, and there was a final elongation step at 72 °C for 1 min [21]. The amplification protocols for *bla*_CIT_, *bla*_ACC_, *bla*_FOX_, *bla*_MOX_, *bla*_DHA_ and *bla*_EBC_ consisted of initial denaturation at 98 °C for 30 s followed by 35 cycles of 98 °C for 5 s, 60 °C for 5 s and 72 °C for 15 s, and there was a final elongation step at 72 °C for 1 min [20]. *E. coli* isolates showing the AmpC phenotype and absence of pAmpC genes were analyzed for mutations in the chromosome-mediated AmpC (*cAmpC*) promoter/attenuator region through the amplification of a 271 bp fragment, as previously reported [22]. That is, we used an amplification protocol consisting of an initial denaturation at 98 °C for 30 s followed by 40 cycles of 98 °C for 5 s, 57 °C for 5 s and 72 °C for 15 s, and there was a final elongation step at 72 °C for 1 min [22]. Amplicons were purified and sequenced by Sanger (by a commercial sequencing facility: Microsynth Seqlab, Göttingen, Germany). The sequences were aligned against the promoter/attenuator region of the *cAmpC* gene of *E. coli* strain ATCC 25922 (GenBank accession number CP009072) using Clustal X in BioEdit software v.7.0. Strains were identified according to the detection of AmpC overproduction or putative low-level AmpC producer modifications, as previously described [24,25]. According to the phenotypical result showing an OXA-48 type, the *bla*_OXA-48_ gene was investigated for carbapenemase production using a PCR protocol with an initial denaturation at 98 °C for 30 s followed by 30 cycles of 98 °C for 5 s, 58 °C for 5 s and 72 °C for 11 s, and there was a final elongation step at 72 °C for 1 min [23]. For each PCR assay, a positive control and a blank control (DNAse-free water) were used. PCR products were visualized under UV after electrophoresis on a 1.5% agarose gel stained with ethidium bromide.

### 2.5. Antimicrobial Minimum Inhibitory Concentration

To establish antimicrobial resistance profile, an antimicrobial minimum inhibitory concentration (MIC) assay was performed on isolates by broth microdilution using commercial plates (Sensititre^TM^ EUVSEC 2 and Sensititre^TM^ EUVSEC 3; ThermoFisher Scientific, Segrate, Milan, Italy) containing 22 antibiotics to be tested following the guidelines of the EUCAST, as described in Decision 2020/1729 EU [26]. The results of the MIC values of each antibiotic were interpreted according to the Epidemiological Cutoff Value (ECOFF) recommended by the European Committee on Antimicrobial Susceptibility Testing, except for sulfamethoxazole, for which the ECOFF was not available [27].

### 2.6. Sample Size Calculation and Statistical Analysis

In order to detect the presence of ESBL-/AmpC-/CP-producing *E. coli* isolates in dogs, a sample size of 100 animals was calculated using WinEpi (http://www.winepi.net/uk/index.htm, accessed on 14 October 2024), considering a population size of 100,000 dogs admitted to the veterinary clinics and a minimum prevalence of 3%.

A binary logistic regression model was used to explore the dependent variable—ESBL-/AmpC-/CP-producing *E. coli* status, which was categorized into distinct positive and the negative groups. The univariable analyses were conducted to test variables for association with ESBL-/AmpC-/CP-producing *E. coli* status, using a binary regression model with one independent variable examined at a time. The investigated variables were sex, age, type of ownership, season of sampling, clinical status, hospitalization, time of sampling in hospitalized dogs, administration of antimicrobial therapy and the administered antimicrobial classes. Variables with a significance level of *p*-value < 0.2 were considered for inclusion in the final model. The final model was constructed ensuring that multicollinearity issues were absent. Only variables with variance inflation factors (VIFs) < 3 were retained in the model. Variables were retained if they substantially improved the model’s fit, as determined by an assessment of the change in deviance. The final model was based on data from 100 cases. Additionally, the Hosmer and Lemeshow test was performed to evaluate the model’s overall fit.

The statistical analysis was conducted using SPSS version 29.0. Variables with a *p*-value ≤ 0.05 were considered statistically significant, and those with a *p*-value ≤ 0.1 were considered to have a tendency towards significance.

## 3. Results

A total of 100 samples were collected. Data regarding the population characteristics of analyzed dogs are summarized in Table 1. Data on sample collection in hospitalized dogs showed that seven dogs were sampled at admission, six were sampled during hospitalization (ranging from one to eleven days after admission) and one was sampled one week following the end of hospitalization.

Overall, 14 (14%; 95% CI: 7.2–20.8%) ESBL-/AmpC-/CP-producing *E. coli* isolates were detected.

The phenotypic characterization showed eleven (11%) ESB-L-, five (5%) AmpC- and one (1%) OXA-48-positive phenotypic tests. Out of the total isolates, more than one positive phenotypic test was observed in 2/14 (14.3%) *E. coli* isolates, and different combinations of positive phenotypic tests and genetic features were observed (Table 2).

Among the fourteen isolates, nine (64.3%) showed an ESBL phenotype, three (21.4%) showed an AmpC phenotype, one (7.1%) showed an ESBL plus AmpC phenotype and one (7.1%) showed an ESBL plus AmpC plus OXA-48 phenotype. The genetic characterization of ESBL-encoding genes showed that *bla*_CTX-M_ was the major gene detected in 9/14 (64.3%) isolates. Among the nine isolates harboring the *bla*_CTX-M_ gene, seven (77.8%) and two (22.2%) strains belonged to group 1 and group 9, respectively. One isolate harboring *bla*_CTX-M-1_ did not show a positive ESBL phenotypic test, but the presence of an AmpC phenotype was observed. ESBL-producing *E. coli* was detected in privately owned healthy and unhealthy dogs. Positivity to *bla*_CMY-2_ was observed in four (28.6%) strains supporting the AmpC phenotype. The promoter/attenuator region analysis of the other AmpC-producing *E. coli* showed the presence of +22 C > T, +26 T > G, +27 A > T and +32 G > A mutations in the attenuator box (Table S2), previously associated with putative low-level AmpC producers [25]. AmpC-producing *E. coli* isolates were detected in both privately owned and dogs from kennels, all unhealthy. In accordance with phenotypic results, the presence of the *bla*_OXA-48_ gene was detected in the ESBL-/AmpC-/CP-producing *E. coli* isolates. The dog harboring the CP-producing *E. coli* strain was an unhealthy puppy from a kennel that was hospitalized, sampled during hospitalization (11 days after admission) and treated with antibiotics.

The overall results of the MIC analysis of the 14 isolates are reported in Table 3, showing the antimicrobial agents tested, the range of dilutions tested for each antimicrobial agent, the number of isolates with MIC results according to the tested dilutions of the antimicrobial agent and the ECOFF reported by EUCAST, which was used to define the presence and total number of resistant isolates [27].

All isolates were resistant to ampicillin, cefotaxime and cefepime, and the majority were resistant to temocillin (12, 85.7%), ceftazidime (10, 71.4%) and tetracycline (10, 71.4%). Resistance to cefoxitin, cefotaxime plus clavulanic acid and ceftazidime plus clavulanic acid were observed only in the AmpC-producing *E. coli* isolates of this study. Overall, seven isolates were resistant to carbapenems based on the ECOFFs reported by EUCAST. More precisely, five (35.7%) and three (21.4%) isolates showed resistance to ertapenem and meropenem, respectively. Only the ESBL-/AmpC-/CP-producing *E. coli* (1, 7.1%) exhibited resistance to both carbapenems, showing the most elevated MIC for ertapenem and the highest MIC value for meropenem, while the other six *E. coli* strains were either resistant to ertapenem only (No. 4) or to meropenem only (No. 2), with lower MIC values. Thirteen (92.9%) isolates showed resistance to three or more different antimicrobial classes and were classified as MDR.

The results of the univariate analysis are reported in Table 1. The final binary logistic regression model adequately fitted the data (Table S3), and two variables were retained. Based on the binary logistic regression analysis, only the variable administration of antimicrobial therapy showed a tendency towards significance, and dogs that received antimicrobial therapy were more likely to test positive for the presence of ESBL-/AmpC-/CP-producing *E. coli* compared to untreated dogs (Table 4).

## 4. Discussion

Multidrug-resistant bacteria, and in particular extended spectrum β-lactamase-resistant *E. coli*, have been reported as an emerging threat for humans and animals worldwide [28]. In the present study, phenotypic and genetic methods were used to investigate the presence of ESBL-/AmpC-/CP-producing *E. coli* in fecal samples from dogs admitted to a Veterinary Teaching Hospital or to other veterinary clinics or kennels in Northern Italy.

The observed 14% presence of ESBL-/AmpC-/CP-producing *E. coli* confirms previous reports in dogs showing prevalences ranging from 3.4% to 25.9% [3,29,30,31]. However, a comparison of the prevalence rates is difficult due to variations in the populations studied, the methodologies employed and other contextual factors that may impact the results [32].

The higher presence of the ESBL phenotype, supported mainly by the presence of the *bla*_CTX-M-1_ gene, confirms previous studies describing ESBL-producing *E. coli* harboring *bla*_CTX-M-1_ as the most common worldwide [33,34]. The presence of the *bla*_TEM_ gene alone in the *E. coli* isolates showing ESBL-positive phenotypic tests likely suggested the ESBL phenotype. However, sequencing of the *bla*_TEM_ gene is needed to confirm this finding because not all TEM-type β-lactamases display the ESBL phenotype [17,20]. The absence of an ESBL phenotype in the strain harboring *bla*_CTX-M_ has been previously reported and may be explained by the presence of AmpC production in the isolate. Indeed, AmpC-producing bacteria may mask an ESBL phenotype [16]. The lower presence of AmpC phenotypes compared to ESBL phenotypes was expected and has been previously reported in dogs [3]. The high frequency of the *bla*_CMY-2_ gene confirms previous studies showing this gene as the most frequently detected in AmpC-producing bacteria [35]. *cAmpC* mutations associated with putative low-level AmpC producers, as found in this study in one positive AmpC phenotype of *E. coli*, have been previously reported [25].

The finding of one CP-producing *E. coli* is of concern, given the threat posed by carbapenemase-producing *Enterobacteriaceae* (CPE) to humans. The prevalence of CP-producing *E. coli* might have been underestimated in our study because we did not use a specific screening method for CPE. Indeed, our screening approach was based on a method to identify ESBL and AmpC β-lactamase producers, using MacConkey agar supplemented with cefotaxime [36,37]. This screening method may be used to detect CP even if it is not considered the method of choice [37,38]. A more accurate estimate of CP-producing bacteria is suggested, using a targeted screening method based on specific substrates or inhibitors to detect carbapenemase activity [37].

The CP-producing *E. coli* supported by the *bla*_OXA-48_ gene identified in this study is in agreement with previous findings, showing that OXA-48-producing isolates have emerged as one of the major CPs [30]. In pets, the *bla*_OXA-48_ gene was firstly discovered in *E. coli* from dogs in Germany in 2013, and afterwards, it was reported in *E. coli* from companion animals in the United States in 2016 [30,39]. In recent years, CP-producing *E. coli* has been identified in dogs worldwide [9,10,40], and the frequency of OXA-48-like-producing bacteria in companion animals highlights the importance of monitoring this resistance mechanism in pets. The elevated MIC result observed for ertapenem in the only confirmed *E. coli* isolate producer of carbapenemases detected in this study confirms previous reports showing that an elevated MIC against carbapenems is commonly recognized as a reliable marker for detecting carbapenemase production [37]. On the other hand, our results showing the in vitro susceptibility to imipenem of the OXA-48 *E. coli* isolate of this study highlight that the use of imipenem as a surrogate to identify carbapenem resistance in clinical microbiology may lead to the misdiagnosis of OXA-48-like-producing bacteria. For these reasons, it is important to employ specific tests not only for screening but also to accurately identify CP-producing bacteria during routine microbiology procedures, in order to improve animal health and minimize their dissemination to humans and the environment [37].

Although the use of carbapenems is not allowed in veterinary medicine in Europe [41,42], at the time of sampling, derogation use was possible for the treatment of companion animals in exceptional cases. Moreover, carbapenem resistance in isolates from dogs may be explained by several factors. Dogs may acquire resistant bacteria from their environment, particularly in settings shared with humans such as homes, parks or veterinary clinics [43]. Our result showing that the dog harboring the CP-producing *E. coli* was a hospitalized puppy from a kennel that was treated with antibiotics suggests CPE acquisition from veterinary hospitals, as previously reported in pets [31].

The presence of MDR *E. coli* with resistance to cephalosporins, ampicillins, tetracyclines and folate antagonist reflects previous studies [44]. Besides the 100% resistance to cefotaxime (14/14), known to be mainly induced by *bla*_CTX-M_, the resistance to cefoxitin, cefotaxime plus clavulanic acid and ceftazidime plus clavulanic acid associated with AmpC-producing *E. coli* isolates of this study is supported by previous studies demonstrating that AmpC enzymes are not inhibited by β-lactamase inhibitors, such as clavulanic acid. Therefore, these inhibitors are unable to neutralize the enzymatic activity of AmpC, which continues to break down β-lactam antibiotics. Additionally, the presence of the *bla*_CMY-2_ gene may have led to an increase in the number of antimicrobials to which the isolate displayed resistance [3].

The high percentage of MDR *E. coli* isolated from dogs is of concern because resistance to multiple critically important antimicrobials poses a serious challenge to veterinary medicine, limits available therapeutic options and increases the risk of these pathogens spreading into the environment, with potential threats for public health [45]. In this respect, the presence of three *E. coli* isolates resistant to colistin, regarded as a last-resort antibiotic for treating infections in humans, is of concern. Further investigations are needed to confirm this finding by genetically characterizing these strains. Recently, colistin resistance has been reported in domestic animals, highlighting the need for intensive surveillance [46,47].

The frequent use of antimicrobials for the treatment of infections in companion animals has been reported to be associated with MDR [48]. Furthermore, treatment with antimicrobials has been reported as a risk factor associated with ESBL-/AmpC-producing *E. coli* in companion animals, supporting our findings showing the tendency of an association of antimicrobial treatment with the fecal carriage of ESBL-/AmpC-/CP-producing *E. coli* [3,49]. Nevertheless, the lack of evidence of a correlation between antibiotic therapy and the development of AMR stains, the limited number of *E. coli* samples, the limited information about the route of administration and duration of antibiotic therapy requires further investigation to confirm this finding. Surprisingly, hospitalization was not observed in our study as a risk factor [3]. Probably, this is due to the low number of hospitalized dogs included in this study or the different management or hygiene protocols among veterinary hospitals [10]. The unhealthy status previously identified as a risk factor for ESBL-/AmpC-producing *E. coli* in pets [50] was not observed in our study. Moreover, our results highlight the possible occurrence of the fecal carriage of ESBL-producing *E. coli* in apparently healthy animals [51,52]. Overall, our results showing the presence of ESBL-/AmpC-/CP-producing *E. coli* in unhealthy dogs treated with antibiotics from kennels, in the absence of hospitalization, highlight the need for continuous surveillance programs in companion animals and not only in veterinary clinics.

This study had some limitations. First, the sample size may not have been sufficiently representative to draw generalizable conclusions for the entire population of dogs. Additionally, this study focused on a limited set of risk factors, neglecting other potential influences such as diet, cohabitation with other animals, or environmental factors. Moreover, bacteria isolated from clinical samples of the unhealthy dogs were not included in this study, and a follow up on hospitalized dogs was not carried in order to analyze the association of outcomes with ESBL-/AmpC-/CP-producing *E. coli* fecal shedding. A significant limitation was the absence of whole-genome sequencing (WGS) analysis, which could have provided a more detailed perspective, allowing for the identification of additional resistance genes that were not considered in this study. Future studies using WGS could also enable a more precise assessment of the presence and distribution of antibiotic-resistant genes in bacteria isolated from domestic animals, especially if living in close contact with humans, in order to define potential transmission among pets, their owners and the environment.

## 5. Conclusions

In conclusion, a One Health perspective concerning AMR spread is needed, considering that ESBL-/AmpC-producing *E. coli* frequently carry antibiotic-resistant genes to clinically important antimicrobial agents that can be transmitted to humans and the environment. It is crucial to establish prevention and control strategies in veterinary practice settings based on antibiotic stewardship. Moreover, the fecal spread of carbapenemase resistance in *E. coli* isolates from domestic dogs, although still limited, emphasizes the need for specific surveillance programs for CP-producing bacteria in companion animals.

## Figures and Tables

**Table 1:** Characteristics of dogs analyzed in this study, including univariate analysis *p*-values for association with the dog’s ESBL-/AmpC-/CP-producing *E. coli* status.

Population Characteristics		No.	No. ESBL-/AmpC-/CP-Producing *E. coli* (%)	*p*-Value
Sex ^1^	Male	46	5 (10.9)	0.388
	Female	53	9 (17)	
Age ^1^	≤2 years	33	5 (15.2)	0.838
	>2 years	66	9 (13.6)	
Type of ownership	Owned	93	11 (11.8)	**0.038**
	Kennel	7	3 (42.9)	
Season of sampling	Winter	41	5 (12.2)	0.500
	Spring	30	3 (10)	
	Summer	16	4 (25)	
	Autumn	13	2 (15.4)	
Clinical status	Healthy	45	5 (11.1)	0.454
	Unhealthy	55	9 (16.4)	
Hospitalization	Yes	14	4 (28.6)	0.102
	No	86	10 (11.6)	
Time of sampling in hospitalized dogs	At admission	7	1 (14.3)	0.256
	After admission	7	3 (42.9)	
Administration of antimicrobial therapy ^2^	Yes	45	10 (21.4)	**0.027**
	No	52	3 (5.6)	
Administered antimicrobial class ^3^	Fluoroquinolones	8	1 (12.5)	0.659
	Β-lactam combination agent	29	7 (24.1)	
	Cephalosporins	2	0	
	Macrolide- nitroimidazole	4	0	
	Tetracyclines	1	0	

^1^ Sex and age were unknown for one dog; ^2^ the administration of antimicrobial therapy was unknown for four dogs; ^3^ the administered antimicrobial class was unknown for three dogs. Values in bold are significant, *p*-value < 0.05.

**Table 2:** Results of phenotypic and genetic characterization of ESBL-/AmpC-/CP-producing *E. coli*.

Phenotypic Characterization	No. (%)	Genes Combination *
ESBL	2 (14.3)	*bla*_CTX-M-1_
	1 (7.1)	*bla*_CTX-M-9_
	3 (21.4)	*bla*_CTX-M-1_, *bla*_TEM_
	1 (7.1)	*bla*_CTX-M-9_, *bla*_TEM_
	2 (14.3)	*bla*_TEM_
AmpC	2 (14.3)	*bla*_CMY-2_
	1 (7.1)	*bla*_CTX-M-1_, *cAmpC* mutations (putative low-level AmpC producer)
ESBL, AmpC	1 (7.1)	*bla*_TEM_, *bla*_CMY-2_
ESBL, AmpC, OXA-48	1 (7.1)	*bla*_CTX-M-1_, *bla*_TEM_, *bla*_CMY-2_, *bla*_OXA-48_

* No positive results for *bla*_SHV_, *bla*_CIT_, *bla*_ACC_, *bla*_FOX_, *bla*_MOX_, *bla*_DHA_, *bla*_EBC_.

**Table 3:** Distribution of MICs among ESBL-/AmpC-/CP-producing *E. coli* isolates (No. = 14). White fields denote range of dilutions tested for each antimicrobial agent, gray fields denote range of dilutions that were not tested for each antimicrobial agents. Vertical lines indicate the ECOFF reported by EUCAST [27].

Antimicrobial Class	Antimicrobial Agent	No. Resistant	No. of Isolates at the Indicated MIC μg/mL																	
			0.0075	0.015	0.03	0.06	0.12	0.25	0.5	1	2	4	8	16	32	64	128	256	512	1024
Β-lactam (penicillins)	AMP	14/14														14				
	TRM	12/14									2	5	3	2	1			1		
Β-lactam (cephalosporins)	FEP	14/14						1		3	1	2	2	2	1	2				
	FOT	14/14											1	5		5	3			
	FOX	5/14									6	1	2		1	1	3			
	TAZ	10/14							2	2			4	3		1		2		
Carbapenems	ETP	5/14		4	5	1		2	1			1								
	IMI	0/14					12	2												
	MERO	3/14			9	2		2	1											
Β-lactam combination agent	F/C	5/14				6	3						2	1	1	1				
	T/C	5/14					5	3	1			1	1	1	2					
Aminoglycoside	AMI	2/14										12			1		1			
	GEN	2/14							10	2					2					
Glycylcyclines	TGC	1/14						8	5	1										
Fluoroquinolones	CIP	8/14		5	1				1	2				5						
Quinolones	NAL	8/14										6		1			7			
Folate antagonist	SMX *	ND												1	1	1				11
	TMP	9/14						3	2						9					
Macrolides	AZI	1/14									1	3	6	3			1			
Phenicols	CHL	4/14											7	3			4			
Polymyxins	COL	3/14								9	2		1		2					
Tetracycline	TET	10/14									4					10				

* No EUCAST ECOFF is available; AMP: ampicillin; TRM: temocillin; FEP: cefepime; FOT: cefotaxime; FOX: cefoxitin; TAZ: ceftazidime; ETP: ertapenem; IMI: imipenem; MERO: meropenem; F/C: cefotaxime/clavulanic acid; T/C: ceftazidime/clavulanic acid; AMI: amikacin; GEN: gentamicin; TGC: tigecycline; CIP: ciprofloxacin; NAL: nalidixic acid; SMX: sulfamethoxazole; TMP: trimethoprim; AZI: Azithromycin; CHL: chloramphenicol; COL: colistin; TET: tetracycline; ND: not determined.

**Table 4:** Binary logistic regression analysis for association of variables with the dog’s ESBL-/AmpC-/CP-producing *E. coli* status.

Variable	*p*-Value	OR (95% CI)
Type of ownership	0.115	0.256 (0.047–1.396)
Administration of antimicrobial therapy	*0.058*	3.868 (0.956–15.647)

Values in italics are tendencies, *p*-value < 0.1; CI—Confidence interval; OR—Odds ratio.

## Data Availability

The data that support the findings of this study are available from the corresponding author upon reasonable request.

## Supplementary Materials

The following supporting information can be downloaded at: https://www.mdpi.com/article/10.3390/ani14233359/s1, Table S1: Primer used in this study; Table S2: Results of the chromosomal AmpC promoter/attenuator region analysis of the AmpC-producing *E. coli* detected in this study; Table S3: Results of the Hosmer and Lemeshow test.

## References

[1] World Health Organization. https://www.who.int/news/item/29-04-2019-new-report-calls-for-urgent-action-to-avert-antimicrobial-resistance-crisis.

[2] Morrison L., Zembower T.R. (2020). Antimicrobial Resistance. Gastrointest. Endosc. Clin. N. Am..

[3] Formenti N., Grassi A., Parisio G., Romeo C., Guarneri F., Birbes L., Pitozzi A., Scali F., Maisano A.M., Boniotti M.B. (2021). Extended-Spectrum-β-Lactamase- and AmpC-Producing *Escherichia coli* in Domestic Dogs: Spread, Characterisation and Associated Risk Factors. Antibiotics.

[4] World Health Organization. https://www.who.int/news-room/fact-sheets/detail/antimicrobial-resistance.

[5] Ljungquist O., Ljungquist D., Myrenås M., Rydén C., Finn M., Bengtsson B. (2016). Evidence of household transfer of ESBL-/pAmpC-producing *Enterobacteriaceae* between humans and dogs—A pilot study. Infect. Ecol. Epidemiol..

[6] Ortega-Paredes D., Haro M., Leoro-Garzón P., Barba P., Loaiza K., Mora F., Fors M., Vinueza-Burgos C., Fernández-Moreira E. (2019). Multidrug-resistant *Escherichia coli* isolated from canine faeces in a public park in Quito, Ecuador. J. Glob. Antimicrob. Resist..

[7] Liu F.L., Kuan N.L., Yeh K.S. (2021). Presence of the Extended-Spectrum-β-Lactamase and Plasmid-Mediated AmpC-Encoding Genes in *Escherichia coli* from Companion Animals-A Study from a University-Based Veterinary Hospital in Taipei, Taiwan. Antibiotics.

[8] Madec J.Y., Haenni M. (2018). Antimicrobial resistance plasmid reservoir in food and food-producing animals. Plasmid.

[9] Yousfi M., Touati A., Mairi A., Brasme L., Gharout-Sait A., Guillard T., De Champs C. (2016). Emergence of Carbapenemase-Producing *Escherichia coli* Isolated from Companion Animals in Algeria. Microb. Drug Resist..

[10] Dazio V., Nigg A., Schmidt J.S., Brilhante M., Mauri N., Kuster S.P., Brawand S.G., Schüpbach-Regula G., Willi B., Endimiani A. (2021). Acquisition and carriage of multidrug-resistant organisms in dogs and cats presented to small animal practices and clinics in Switzerland. J. Vet. Intern. Med..

[11] Roscetto E., Varriale C., Galdiero U., Esposito C., Catania M.R. (2021). Extended-Spectrum Beta-Lactamase-Producing and Carbapenem-Resistant *Enterobacterales* in Companion and Animal-Assisted Interventions Dogs. Int. J. Environ. Res. Public Health.

[12] Marchetti L., Buldain D., Gortari Castillo L., Buchamer A., Chirino-Trejo M., Mestorino N. (2021). Pet and Stray Dogs as Reservoirs of Antimicrobial-Resistant *Escherichia coli*. Int. J. Microbiol..

[13] Guerra B., Fischer J., Helmuth R. (2014). An emerging public health problem: Acquired carbapenemase-producing microorganisms are present in food-producing animals, their environment, companion animals and wild birds. Vet. Microbiol..

[14] Usmael B., Abraha B., Alemu S., Mummed B., Hiko A., Abdurehman A. (2022). Isolation, antimicrobial susceptibility patterns, and risk factors assessment of non-typhoidal *Salmonella* from apparently healthy and diarrheic dogs. BMC Vet. Res..

[15] Singhal N., Kumar M., Kanaujia P.K., Virdi J.S. (2015). MALDI-TOF mass spectrometry: An emerging technology for microbial identification and diagnosis. Front. Microbiol..

[16] EUCAST Guidelines for Detection of Resistance Mechanisms and Specific Resistances of Clinical and/or Epidemiological Importance (V 2.0 July 2017). https://www.eucast.org/fileadmin/src/media/PDFs/EUCAST_files/Resistance_mechanisms/EUCAST_detection_of_resistance_mechanisms_170711.pdf.

[17] Monstein H.J., Ostholm-Balkhed A., Nilsson M.V., Nilsson M., Dornbusch K., Nilsson L.E. (2007). Multiplex PCR amplification assay for the detection of *bla*_SHV_, *bla*_TEM_ and *bla*_CTX-M_ genes in *Enterobacteriaceae*. APMIS.

[18] Saladin M., Cao V.T., Lambert T., Donay J.L., Herrmann J.L., Ould-Hocine Z., Verdet C., Delisle F., Philippon A., Arlet G. (2002). Diversity of CTX-M beta-lactamases and their promoter regions from *Enterobacteriaceae* isolated in three Parisian hospitals. FEMS Microbiol. Lett..

[19] Jiang X., Ni Y., Jiang Y., Yuan F., Han L., Li M., Liu H., Yang L., Lu Y. (2005). Outbreak of infection caused by *Enterobacter cloacae* producing the novel VEB-3 beta-lactamase in China. J. Clin. Microbiol..

[20] Dallenne C., Da Costa A., Decré D., Favier C., Arlet G. (2010). Development of a set of multiplex PCR assays for the detection of genes encoding important beta-lactamases in *Enterobacteriaceae*. J. Antimicrob. Chemother..

[21] Rehman M.A., Hasted T.L., Persaud-Lachhman M.G., Yin X., Carrillo C., Diarra M.S. (2019). Genome Analysis and Multiplex PCR Method for the Molecular Detection of Coresistance to Cephalosporins and Fosfomycin in *Salmonella enterica* Serovar Heidelberg. J. Food Prot..

[22] Corvec S., Prodhomme A., Giraudeau C., Dauvergne S., Reynaud A., Caroff N. (2007). Most *Escherichia coli* strains overproducing chromosomal AmpC beta-lactamase belong to phylogenetic group A. J. Antimicrob. Chemother..

[23] European Centre for Disease Prevention and Control (2019). Laboratory Manual for Carbapenem and Colistin Resistance Detection and Characterisation for the Survey of Carbapenem-and/or Colistin-Resistant Enterobacteriaceae—Version 2.0.

[24] Peter-Getzlaff S., Polsfuss S., Poledica M., Hombach M., Giger J., Böttger E.C., Zbinden R., Bloemberg G.V. (2011). Detection of AmpC beta-lactamase in *Escherichia coli*: Comparison of three phenotypic confirmation assays and genetic analysis. J. Clin. Microbiol..

[25] Coolen J.P.M., den Drijver E.P.M., Verweij J.J., Schildkraut J.A., Neveling K., Melchers W.J.G., Kolwijck E., Wertheim H.F.L., Kluytmans J.A.J.W., Huynen M.A. (2021). Genome-wide analysis in *Escherichia coli* unravels a high level of genetic homoplasy associated with cefotaxime resistance. Microb. Genom..

[26] Commission Implementing Decision (EU) 2020/1729 of 17 November 2020 on the Monitoring and Reporting of Antimicrobial Resistance in Zoonotic and Commensal Bacteria and Repealing Implementing Decision 2013/652/EU (Notified under Document C(2020) 7894). https://eur-lex.europa.eu/legal-content/EN/TXT/PDF/?uri=CELEX:32020D1729.

[27] European Committee on Antimicrobial Susceptibility Testing Data from the EUCAST MIC Distribution Website. https://www.eucast.org.

[28] Perestrelo S., Amaro A., Brouwer M.S.M., Clemente L., Ribeiro Duarte A.S., Kaesbohrer A., Karpíšková R., Lopez-Chavarrias V., Morris D., Prendergast D. (2023). Building an International One Health Strain Level Database to Characterise the Epidemiology of AMR Threats: ESBL-AmpC Producing *E. coli* as an Example-Challenges and Perspectives. Antibiotics.

[29] Huber H., Zweifel C., Wittenbrink M.M., Stephan R. (2013). ESBL-producing uropathogenic *Escherichia coli* isolated from dogs and cats in Switzerland. Vet. Microbiol..

[30] Liu X., Liu H., Li Y., Hao C. (2016). High Prevalence of β-lactamase and Plasmid-Mediated Quinolone Resistance Genes in Extended-Spectrum Cephalosporin-Resistant *Escherichia coli* from Dogs in Shaanxi, China. Front. Microbiol..

[31] Haenni M., Boulouis H.J., Lagrée A.C., Drapeau A., Va F., Billet M., Châtre P., Madec J.Y. (2022). *Enterobacterales* high-risk clones and plasmids spreading *bla*_ESBL/AmpC_ and *bla*_OXA-48_ genes within and between hospitalized dogs and their environment. J. Antimicrob. Chemother..

[32] Ewers C., Bethe A., Semmler T., Guenther S., Wieler L.H. (2012). Extended-spectrum β-lactamase-producing and AmpC-producing *Escherichia coli* from livestock and companion animals, and their putative impact on public health: A global perspective. Clin. Microbiol. Infect..

[33] Peirano G., Pitout J.D.D. (2019). Extended-Spectrum β-Lactamase-Producing *Enterobacteriaceae*: Update on Molecular Epidemiology and Treatment Options. Drugs.

[34] Benavides J.A., Salgado-Caxito M., Opazo-Capurro A., González Muñoz P., Piñeiro A., Otto Medina M., Rivas L., Munita J., Millán J. (2021). ESBL-Producing *Escherichia coli* Carrying CTX-M Genes Circulating among Livestock, Dogs, and Wild Mammals in Small-Scale Farms of Central Chile. Antibiotics.

[35] Melo L.C., Oresco C., Leigue L., Netto H.M., Melville P.A., Benites N.R., Saras E., Haenni M., Lincopan N., Madec J.Y. (2018). Prevalence and molecular features of ESBL/pAmpC-producing *Enterobacteriaceae* in healthy and diseased companion animals in Brazil. Vet. Microbiol..

[36] Schmiedel J., Falgenhauer L., Domann E., Bauerfeind R., Prenger-Berninghoff E., Imirzalioglu C., Chakraborty T. (2014). Multiresistant extended-spectrum β-lactamase-producing *Enterobacteriaceae* from humans, companion animals and horses in central Hesse, Germany. BMC Microbiol..

[37] Silva J.M.D., Menezes J., Marques C., Pomba C.F. (2022). Companion Animals–An Overlooked and Misdiagnosed Reservoir of Carbapenem Resistance. Antibiotics.

[38] European Food Safety Authority (EFSA), European Centre for Disease Prevention and Control (ECDC) (2024). The European Union summary report on antimicrobial resistance in zoonotic and indicator bacteria from humans, animals and food in 2021–2022. EFSA J..

[39] Stolle I., Prenger-Berninghoff E., Stamm I., Scheufen S., Hassdenteufel E., Guenther S., Bethe A., Pfeifer Y., Ewers C. (2013). Emergence of OXA-48 carbapenemase-producing *Escherichia coli* and *Klebsiella pneumoniae* in dogs. J. Antimicrob. Chemother..

[40] Alba P., Taddei R., Cordaro G., Fontana M.C., Toschi E., Gaibani P., Marani I., Giacomi A., Diaconu E.L., Iurescia M. (2021). Carbapenemase IncF-borne *bla*_NDM-5_ gene in the *E. coli* ST167 high-risk clone from canine clinical infection, Italy. Vet. Microbiol..

[41] European Medicines Agency (EMA) (2019). Categorisation of Antibiotics in the European Union (London, UK: European Medicines Agency). http://www.ema.europa.eu/en/annual-report-2019/antimicrobial-resistance.html.

[42] Schmerold I., van Geijlswijk I., Gehring R. (2023). European regulations on the use of antibiotics in veterinary medicine. Eur. J. Pharm. Sci..

[43] Damborg P., Broens E.M., Chomel B.B., Guenther S., Pasmans F., Wagenaar J.A., Weese J.S., Wieler L.H., Windahl U., Vanrompay D. (2016). Bacterial Zoonoses Transmitted by Household Pets: State-of-the-Art and Future Perspectives for Targeted Research and Policy Actions. J. Comp. Pathol..

[44] Wedley A.L., Dawson S., Maddox T.W., Coyne K.P., Pinchbeck G.L., Clegg P., Nuttall T., Kirchner M., Williams N.J. (2017). Carriage of antimicrobial resistant *Escherichia coli* in dogs: Prevalence, associated risk factors and molecular characteristics. Vet. Microbiol..

[45] Walther B., Tedin K., Lübke-Becker A. (2017). Multidrug-resistant opportunistic pathogens challenging veterinary infection control. Vet. Microbiol..

[46] Menezes J., Moreira da Silva J., Frosini S.M., Loeffler A., Weese S., Perreten V., Schwarz S., Telo da Gama L., Amaral A.J., Pomba C. (2022). *mcr-1* colistin resistance gene sharing between *Escherichia coli* from cohabiting dogs and humans, Lisbon, Portugal, 2018 to 2020. Eurosurveillance.

[47] Moon D.C., Mechesso A.F., Kang H.Y., Kim S.J., Choi J.H., Kim M.H., Song H.J., Yoon S.S., Lim S.K. (2020). First Report of an *Escherichia coli* Strain Carrying the Colistin Resistance Determinant *mcr-1* from a Dog in South Korea. Antibiotics.

[48] Choi J.H., Ali M.S., Moon B.Y., Kang H.Y., Kim S.J., Song H.J., Mechesso A.F., Moon D.C., Lim S.K. (2023). Prevalence and Characterization of Extended-Spectrum β-Lactamase-Producing *Escherichia coli* Isolated from Dogs and Cats in South Korea. Antibiotics.

[49] Pomba C., Rantala M., Greko C., Baptiste K.E., Catry B., van Duijkeren E., Mateus A., Moreno M.A., Pyörälä S., Ružauskas M. (2017). Public health risk of antimicrobial resistance transfer from companion animals. J. Antimicrob. Chemother..

[50] Ortiz-Díez G., Mengíbar R.L., Turrientes M.C., Artigao M.B., Gallifa R.L., Tello A.M., Pérez C.F., Santiago T.A. (2023). Prevalence, incidence and risk factors for acquisition and colonization of extended-spectrum beta-lactamase- and carbapenemase-producing *Enterobacteriaceae* from dogs attended at a veterinary hospital in Spain. Comp. Immunol. Microbiol. Infect. Dis..

[51] Schmidt V.M., Pinchbeck G.L., Nuttall T., McEwan N., Dawson S., Williams N.J. (2015). Antimicrobial resistance risk factors and characterisation of faecal *E. coli* isolated from healthy Labrador retrievers in the United Kingdom. Prev. Vet. Med..

[52] Werhahn Beining M., Hartmann M., Luebke-Becker A., Guenther S., Schaufler K., Hille K., Kreienbrock L. (2023). Carriage of Extended Spectrum Beta Lactamase-Producing *Escherichia coli*: Prevalence and Factors Associated with Fecal Colonization of Dogs from a Pet Clinic in Lower Saxony, Germany. Animals.

