# Supplementary material for: Fecal Carriage and Risk Factors Associated with Extended-Spectrum β-Lactamase-/AmpC-/Carbapenemase-Producing Escherichia coli in Dogs from Italy
Source: Animals (Basel). 2024 Nov 21;14(23):3359. doi: 10.3390/ani14233359 (PMC11640086; doi:10.3390/ani14233359)
Supplement: Supplementary file 1 [file animals-14-03359-s001.zip › Facchin et al._Table S1.pdf]

Table S1. Primers used in this study.

Target	Primer	Sequence 5′-3′	Amplicon size (bp)	Reference
CTX-M	$bla_{\text{CTX-M}}$ F<br>$bla_{\text{CTX-M}}$ R	ATGTGCAGYACCAGTAARGTKATGGC<br>TGGGTRAARTARGTSACCAGAAYCAGCGG	593	[17]
CTX-M-1 group	$bla_{\text{CTX-M-1}}$ F<br>$bla_{\text{CTX-M-1}}$ R	GGTTAAAAAATCACTGCGYC<br>TYGGTGACGATTTTAGCCGC	865	[18]
CTX-M-9 group	$bla_{\text{CTX-M-9}}$ F<br>$bla_{\text{CTX-M-9}}$ R	ATGGTGACAAAGAGAGTGCA<br>CCCTTCGGCGATGATTCTC	865	[19]
TEM	bla_{TEM} F<br>bla_{TEM} R	TCGCCGCATACACTATTCTCAGAATGA<br>ACGCTCACCGGCTCCAGATTTAT	445	[17]
SHV	bla_{SHV} F<br>bla_{SHV} R	ATGCGTTATATTCGCCTGTG<br>TGCTTTGTTATTCGGGCCAA	747	[17]
CMY-2	$bla_{\text{CMY-2}}$ F<br>$bla_{\text{CMY-2}}$ R	TGGCCGTTGCCGTTATCTAC<br>CCCGTTTTATGCACCCATGA	820	[21]
CIT	MultiCaseCIT_for<br>MultiCaseCIT_rev	CGAAGAGGCAATGACCAGAC<br>ACGGACAGGGTTAGGATAGY	538	[20]
ACC	MultiCaseACC_for<br>MultiCaseACC_rev	CACCTCCAGCGACTTGTTAC<br>GTTAGCCAGCATCACGATCC	346	[20]
FOX	MultiCaseFOX_for<br>MultiCaseFOX_rev	CTACAGTGCGGGTGGTTT<br>CTATTTGCGGCCAGGTGA	162	[20]
MOX	MultiCaseMOX_for<br>MultiCaseMOX_rev	GCAACAACGACAATCCATCCT<br>GGGATAGGCGTAACTCTCCCAA	895	[20]
DHA	MultiCaseDHA_for<br>MultiCaseDHA_rev	TGATGGCACAGCAGGATATTC<br>GCTTTGACTCTTTCGGTATTCG	997	[20]
EBC	MultiCaseEBC_for<br>MultiCaseEBC_rev	CGGTAAAGCCGATGTTGCG<br>AGCCTAACCCCTGATACA	683	[20]
cAmpC promoter/ attenuator region	AmpC AB1<br>AmpC-2	GATCGTTCTGCCGCTGTG<br>GGGCAGCAAATGTGGAGCAA	271	[22]
OXA-48	$bla_{\text{OXA-48}}$ F<br>$bla_{\text{OXA-48}}$ R	TTGGTGGCATCGATTATCGG-<br>GAGCACTTCTTTTGTGATGGC	744	[23]
