# Supplementary material for: Fecal Carriage and Risk Factors Associated with Extended-Spectrum β-Lactamase-/AmpC-/Carbapenemase-Producing Escherichia coli in Dogs from Italy
Source: Animals (Basel). 2024 Nov 21;14(23):3359. doi: 10.3390/ani14233359 (PMC11640086; doi:10.3390/ani14233359)
Supplement: Supplementary file 1 [file animals-14-03359-s001.zip › Facchin et al._Table S2.pdf]

Table S2. Results of the chromosomal AmpC promoter/attenuator region analysis of the AmpC-producing *E. coli* detected in this study.

Position within the AmpC promoter/attenuator regions*		ATCC 25922 (accession number: CP009072)	152/1 (AmpC-producing E. coli detected in this study)
Displaced -35 box	-42	C	
-35 box	-32	T	
	-28	G	
	-26.1	-	
	-23	T	
	-20.1	-	
Displaced 10 box	-18	G	
Spacer	-15.2	-	
	-15.1	-	
	-15	G	
-10 box	-11	C	
Attenuator	+17	C	
	+21.1	-	
	+22	C	T
	+23	G	
	+24	C	
	+26	T	G
	+27	A	T
	+28	T	
	+30	G	
	+32	G	A
	+34	G	
	+35	C	
	+36	C	
	+37	G	

* Descriptions of functional elements are used as reported by [24,25].
