# Supplementary material for: Fecal Carriage and Risk Factors Associated with Extended-Spectrum β-Lactamase-/AmpC-/Carbapenemase-Producing Escherichia coli in Dogs from Italy
Source: Animals (Basel). 2024 Nov 21;14(23):3359. doi: 10.3390/ani14233359 (PMC11640086; doi:10.3390/ani14233359)
Supplement: Supplementary file 1 [file animals-14-03359-s001.zip › Facchin et al._Table S3.pdf]

Table S3. Results of the Hosmer and Lemeshow test.

Chi-square	*p*-value	df
0.019	0.891	**1**

df: degree of freedom
